# Antigenic Proteins Involved in Occupational Rhinitis and Asthma Caused by Obeche Wood (*Triplochiton Scleroxylon*)

**DOI:** 10.1371/journal.pone.0053926

**Published:** 2013-01-22

**Authors:** Ana Aranda, Paloma Campo, Arantxa Palacin, Inmaculada Doña, Cristina Gomez-Casado, Luisa Galindo, Araceli Díaz-Perales, Miguel Blanca

**Affiliations:** 1 Allergy Research Laboratory, Carlos Haya Hospital, Málaga, Spain; 2 Unidad de Gestión Clínica Allergy, Carlos Haya Hospital, Málaga, Spain; 3 Biotechnology Department, Center for Plant Biotechnology and Genomics, Pozuelo de Alarcon, Madrid, Spain; University of South Florida College of Medicine, United States of America

## Abstract

**Background:**

Obeche wood dust is a known cause of occupational asthma where an IgE-mediated mechanism has been demonstrated.

**Objective:**

To characterize the allergenic profile of obeche wood dust and evaluate the reactivity of the proteins by *in vitro*, *ex vivo* and *in vivo* assays in carpenters with confirmed rhinitis and/or asthma

**Materials and methods:**

An in-house obeche extract was obtained, and two IgE binding bands were purified (24 and 12 kDa) and sequenced by N-terminal identity. Specific IgE and IgG, basophil activation tests and skin prick tests (SPTs) were performed with whole extract and purified proteins. CCD binding was analyzed by ELISA inhibition studies.

**Results:**

Sixty-two subjects participated: 12 with confirmed occupational asthma/rhinitis (ORA+), 40 asymptomatic exposed (ORA−), and 10 controls. Of the confirmed subjects, 83% had a positive SPT to obeche. There was a 100% recognition by ELISA in symptomatic subjects vs. 30% and 10% in asymptomatic exposed subjects and controls respectively (p<0.05). Two new proteins were purified, a 24 kDa protein identified as a putative thaumatin-like protein and a 12 kDa gamma-expansin. Both showed allergenic activity *in vitro*, with the putative thaumatin being the most active, with 92% recognition by ELISA and 100% by basophil activation test in ORA+ subjects. Cross-reactivity due to CCD was ruled out in 82% of cases.

**Conclusions:**

Two proteins of obeche wood were identified and were recognized by a high percentage of symptomatic subjects and by a small proportion of asymptomatic exposed subjects. Further studies are required to evaluate cross reactivity with other plant allergens.

## Introduction

Wood is a known sensitizer widely extended in the construction and furniture industries worldwide [1], [2]. Exposure to wood dust has been associated with the risk of developing asthma, an increase in airway hyperresponsiveness and decreased FEV_1_ [3], [4], even in apprentices [5]. The obeche (*Triplochiton scleroxylon*) is a tree from West Africa belonging to the *Sterculiaceace* family [6]. Several cases of occupational asthma due to inhalation of obeche dust have been reported, with positive skin prick tests (SPTs), specific IgE to wood extracts and positive bronchial challenges suggesting an IgE-mediated mechanism [6]–[10]. However, there is no standardized extract for accurate diagnosis and the nature of the IgE-binding components in obeche is not fully known. Two reports [6]–[9] first described IgE binding bands by means of SDS-PAGE and immunodetection assays. In the study by Quirce *et al*, allergic subjects mostly recognized a 28 kDa band [6], while other bands of 64, 57.8, and 17 kDa were recognized with less intensity. In addition, IgE reactivity was reduced only by 8.6% when a deglycosylated extract was used, which indicates a non-carbohydrate nature of these IgE binding proteins. The same 28 and 17 kDa reactive components were also found in two other studies [9], [11]. One of these, that of Vidal *et al*, described the presence of the 28 kDa protein in both obeche and in fruit extracts (*Cyphomandra betacea Sendth* or tamarillo), and this protein was recognized *in vitro* by a carpenter with occupational asthma due to obeche who suffered anaphylaxis after ingestion of tamarillo [11]. However, no further experiments were conducted to identify these components. Later on, a high molecular weight class I chitinase was described (Trip s1), and still remains the only allergen identified so far [10]. This 38 kDa protein shares a high molecular similarity with Prs a 1 (avocado allergen) and with Hev b 6, and cross reactivity has been demonstrated in a small group of sensitized subjects [10], [12]. However, this allergen was not recognized by most subjects in the aforementioned studies [6], [9], [11], so its relevance in other populations remains unknown. Thus, the allergenic content of obeche wood needs to be analyzed in detail.

The aim of this study was to further characterize the allergenic profile of obeche wood dust. We also evaluated the reactivity of the potentially allergenic proteins by *in vitro*, *ex vivo* and *in vivo* assays in a well-characterized population of carpenters with confirmed rhinitis and/or asthma due to obeche wood exposure and two control populations.

## Materials and Methods

### Production of in-house obeche extract

Obeche wood dust was extracted with phosphate-buffered saline (PBS) buffer for 1 h at 4°C, centrifuged at 10000× g for 30 min at 4°C. The supernatant was dialysed (cut-off point, 3.5 kDa) against H_2_O and freeze-dried. The protein concentration was quantified according to the method of Bradford (Pierce Biotechnology, In. Rockford, USA).

### Study population

This study involved carpenters and carpentry apprentices with respiratory symptoms (nasal and/or bronchial) due to occupational exposure to obeche wood, and with a diagnosis of occupational rhinitis/asthma confirmed by specific inhalation challenge with the in-house obeche extract at 1 mg/ml. Nasal challenges were performed according to published methods [13], and responses were monitored by acoustic rhinometry, visual analogue scale and symptoms score. Bronchial challenges were performed using a DeVilbiss nebulizer and responses were monitored by serial spirometry [14]. Nasal and bronchial challenges were performed with the same extract in 5 controls showing negative responses. A group of asymptomatic exposed subjects, with negative SPT to obeche extract who worked in the same factories or school as the symptomatic subjects were invited to participate, and a number of them were randomly selected for the study. A group of non-exposed asymptomatic subjects was also recruited as a control group. All participants completed an occupational questionnaire, as described [15]. Spirometry was performed using a Spirobank spirometer (RDSM, Hasselt, Belgium) following the guidelines [16]. Skin prick tests (SPTs) (ALK-Abelló, Spain) were performed [17] with a battery of common aeroallergens that included grass pollen, trees, dust mites, molds, dog/cat dander and latex. Also, SPT with thaumatin-containing food extracts (banana, peach, hazelnut, chestnut, kiwi, apple and melon, ALK-Abelló, Spain) were performed. Written informed consent was obtained from all subjects and the ethical committee of our institution approved the study.

### Isolation of allergens

Obeche extract was fractionated by anion-exchange chromatography on a Bio-Scale™ Mini Macro-Prep® High Q column (BioRad, Hercules, CA, USA) equilibrated 20 mM ethalonamine, pH 9, and eluted with 1 M NaCl in the same buffer. Retained fractions were identified by SDS-PAGE and immunodetection with a serum pool from confirmed obeche-sensitised patients. The non-retained material was separated by RP-HPLC on Europa protein C4 column (25×0.7 mm; particle size 5 µm; Teknokroma, Barcelona, Spain). Elution was performed with a linear gradient of acetonitrile in 0.1% (v/v) trifluoroacetic acid (0–10% for 15 min and 10–100% for 150 min, at a flow rate of 0.5 ml/min). Peaks were processed along with the retained ion exchange fractions. The purified proteins were quantified by a commercial bicinchoninic acid test (Pierce, Cheshire, UK) and their purity was tested by SDS-PAGE, N-terminal amino acid sequencing with an Applied Biosystems 477A gas-phase sequencer (Applied Biosystems, CA, USA), and mass spectrophotometric analysis with a Biflex III Spectrometer (Bruker-Franzen Analytik, Bremen, Germany), using standard methods.

### SDS-PAGE and immunodetection

Obeche extract (30 µg) and purified proteins (5 µg) were separated on SDS-PAGE [18]. Proteins were electrotransferred to polyvinylidene difluoride membranes. Blocked membranes (Blocking solution, Sigma, St.Louis, USA) were incubated with a pool of sera from symptomatic exposed subjects with positive specific IgE to obeche (1∶5 dilution). The IgE binding was revealed with anti-human IgE-peroxidase conjugate (Biosource, Camarillo, CA, USA; 1∶3000 dilution) and chemiluminiscence (Amersham Supersignal West Pico Chemiluminutoescent Substrate, Rockford, USA). Further, blocked membranes were incubated with different polyclonal antibodies and then treated with goat anti-rabbit IgG-peroxidase conjugate (Dako, Carpinteria, CA, USA; 1∶10000 dilution) and revealed by means of chemiluminiscence.

### Skin prick tests with obeche extract and purified allergens

SPTs with the in-house obeche dust extract (1 mg/ml protein in a 1∶1 [v/v] PBS buffer/glycerol solution) or with purified proteins (20 µg/ml) were carried out [17]. Histamine dihydrochloride (10 mg/ml) and PBS buffer/glycerol solution served as positive and negative controls, respectively. Ten non-exposed asymptomatic subjects were tested with this obeche extract and purified proteins, and the responses were all negative.

### Specific immunoglobulin E and G determination

Specific IgE and IgG antibodies to obeche wood dust extract were determined in each individual serum in triplicate by direct ELISA method [19]. Obeche extract was tested at 20 µg/ml and purified proteins at 5 µg/ml as solid phase, and serum at 1∶10 dilution for measurement of IgE and at 1∶50 dilution for measurement of IgG. Blocking solution was used as a negative control. Specific IgE levels greater than 0.19 OD units and specific IgG levels greater than 0,25 OD units (mean [OD]+3× S.D. to blocking) were considered positive. Sera from subjects with confirmed occupational rhinitis and/or asthma were selected to analyze the IgE binding activity of the purified proteins as solid phase following the same protocol described above.

### ELISA inhibition studies with CCD determinants

Sera from subjects with confirmed occupational asthma/rhinitis were pre-incubated with different inhibitors (in-house obeche extract and bromelain, Sigma, St. Louis, USA) at final concentrations of 100, 10 and 1 µg/ml at room temperature for 3 h [19]. Later on, the inhibitor mixtures (including sera with no inhibitor as a positive control) were added to plates coated with obeche extract a 20 µg/ml and incubated at 37°C overnight. The assay was completed by incubating with rabbit anti-human IgE antibody (1∶3000; DAKO A/S, Denmark). IgE binding was detected using o-phenylenediamine tablets (OPD, DAKO) and the reaction was stopped by adding 2 M chloric acid. The absorbance (OD) in each well was measured at 490 nm. The percentage inhibition of IgE binding was calculated using the following formula: % inhibition = (serum not inhibited-serum inhibited/serum not inhibited) ×100.

### Basophil activation test

The basophil activation test (BAT) was performed as described [20], using different concentrations of obeche extract (50, 25, 12.5 and 6.25 µg/ml) and of purified proteins (10, 5 and 2,5 µg/ml). The optimal concentrations of extract and purified proteins were chosen based on dose-response curves and cytotoxicity studies. Results were considered as positive when the stimulation index (SI), calculated as the ratio between the percentage of degranulated basophils with the allergens and the negative control, was >2 in at least one of the dilutions.

### Statistical analysis

Differences between groups were compared by the Student t test for continuous variables and by Chi square test for categorical variables using the SPSS software package (SPSS Corporation, Chicago, IL); p values<0.05 were considered statistically significant.

## Results

### Clinical characteristics of study population

Sixty-two subjects participated in the study:

1. Subjects with confirmed occupational rhinitis and/or asthma (n = 12). These subjects had a diagnosis of either occupational rhinitis (n = 7) or asthma (n = 5) as confirmed by specific nasal or bronchial challenges, all having immediate isolated nasal or bronchial responses. These subjects comprised the occupational rhinitis/asthma group (ORA+).
2. Subjects who were occupationally exposed to obeche wood without any respiratory symptoms and with a negative SPT to obeche extract (n = 40). These subjects comprised the non occupational rhinitis/asthma (ORA−) group.
3. Control group of non-exposed, asymptomatic subjects (CG, n = 10).

The characteristics of the ORA+ subjects were compared to the ORA- group and the control group (see **Table 1**). ORA+ subjects were older and had a significantly longer exposure time when compared with the asymptomatic group (p<0.05). ORA+ subjects had higher total IgE levels compared to ORA− and CG although this difference was not significant. The percentage of smokers and the FEV_1_ were similar in all groups. Both ORA+ and ORA− subjects had a high percentage of positive SPT to pollen (ORA+:75%, 67% grass pollen; ORA−: 49%, 60% grass pollen).

**Table 1:** Demographic and clinical characteristics of subjects with confirmed occupational rhinitis/asthma (ORA+) *vs.* asymptomatic exposed (ORA−) and controls (CG). SPT: skin prick test; SD: standard deviation; *: p<0.05.

	ORA+	ORA−	CG
	n = 12	n = 40	n = 10
Age, mean (SD)*	42,3 (13)	20,05 (2,8)	36,33 (7,2)
Sex, M/F*	10/2	40/0	2/8
**Smokers, (%)**	41,7%	29,3%	30%
**Total IgE, mean (range)**	250 (54–688)	192 (3–1270)	64 (3–270)
Mean exposure to wood, months*	238	42	0
**Mean FEV_1_, %**	93%	94%	95%
**Positive SPTs to pollen, %**	75% (67% grass)	49% (60% grass)	30% (33% grass)

### Measurement of specific IgE antibodies against obeche wood

**Figure 1:**
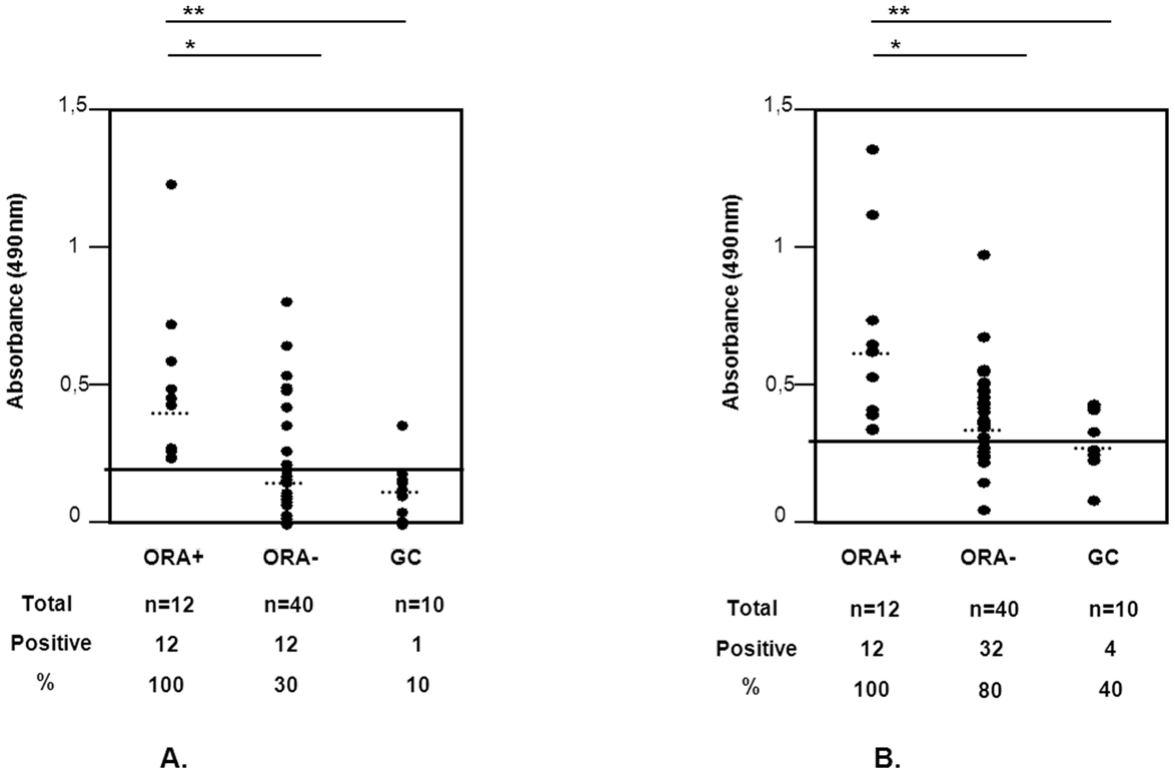
Measurement of specific antibodies to obeche wood dust. Means are represented by dotted lines and a horizontal line indicates cut-off value. *p<0.05 symptomatic vs asymptomatics; **p<0.05 symptomatic vs controls. **1A**. Levels of specific IgE in ORA+, ORA− and CG subjects; **1B**. Levels of specific IgG in ORA+, ORA− and CG subjects.

Specific IgE antibodies to obeche wood were measured by ELISA in all ORA+, ORA− and CG subjects. As shown in **Figure 1a**, 100% of the ORA+ subjects had specific IgE antibodies against obeche extract, with the highest mean OD of the three groups (solid line shows the positivity cut-off point, dotted lines show mean OD for each group). On the other hand, only 30% of the ORA− subjects had specific IgE antibodies (p<0.05 ORA+ *vs.* ORA−). The control group of unexposed subjects showed the lowest mean value, below the cut-off point of positivity, with only one serum showing a positive value. Thus, detection of specific IgE antibodies was significantly higher (p<0.05) in symptomatic subjects as compared to asymptomatic subjects and controls. **Figure 1b** shows the measurement of specific IgG. We observed 100% positive results in ORA+ subjects and 80% in ORA− subjects. However, 40% of unexposed subjects also showed positive results, with the mean value of this group below the threshold of positivity (p<0.05).

### In-house obeche extract showed two principal IgE binding bands

**Figure 2:**
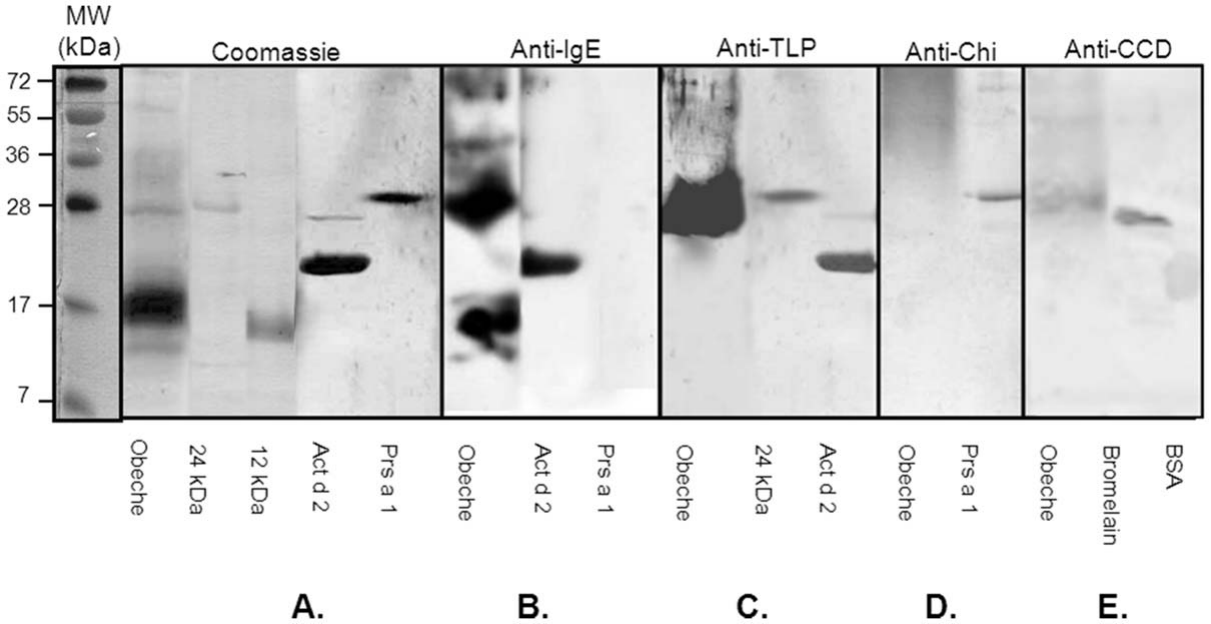
Immunodetection of proteins in obeche wood dust. **2A**. *In-house* obeche wood dust extract (40 µg; Obeche), purified protein (5 µg; 24 kDa; 12 kDa) or purified allergens Act d 2 and Prs a 1 (5 µg) were separated by SDS-PAGE and stained with Coomassie blue (Coomassie). Replicas from the extract were electrotransferred and incubated with serum pool of selected exposure subjects (anti-IgE) (**2B**) or with antibodies produced against chestnut thaumatin-like protein (anti-TLP) (**2C**), against chestnut chitinases (anti-Ch) (**2D**) or anti N-glycan antibodies (anti-CCD) with bromelain and bovine serum albumin (BSA) as positive and negative controls (**2E**).

To better characterize the molecular basis of obeche sensitization, the in-house extract was separated by SDS-PAGE and stained with Coomassie (**Figure 2A**). Replicas were electrotransferred and incubated with a pool of sera of sensitized subjects. Two principal bands seemed to be recognized with the highest intensity by serum pool: a 24 kDa and a band with an apparent molecular weight of 12 kDa. In order to identify the nature of these IgE-binding bands, their purification was investigated through different ionic exchange chromatographic techniques. The high molecular weight protein was the most difficult to purify since it was extremely unstable. For this reason, the amount of this purified protein was very small and therefore it was not possible to obtain enough protein to analyze its allergic activity *in vivo* by skin prick test in all subjects. However, the two proteins were purified in sufficient quantity to demonstrate their allergenic activity by *in vitro* (ELISA and BAT) assays. Their purity was checked sequencing their N-terminal amino acids and analyzing their real molecular weight by mass spectrometry.

### A gamma-expansin was identified as new allergen of obeche

The protein with the lowest molecular weight (12 kDa) presented 11642.5 Da by mass spectrometry, and its N-terminal amino acid was sequenced and rendered IQGTATFYTPPYVPS. This sequence matched in homology with gamma-expansins of alder (*Alnus glutinasa*; GU06293.1) and of lemon tree (*Citrus jambluri*; AF015782). Members of the same protein family have been associated with grass pollen allergy (group 1 of grass allergens) although their sequence identity with the new allergen is poor [21].

### The 24 kDa IgE binding protein could belong to the thaumatin-like family

The highest protein showed 23590.3 Da and its N-terminal corresponded to AQITVTYNGQNG. However, the number of residues was not enough to find identity with known sequences deposited in data banks. For this reason, SDS-PAGE bands were excised and in-gel trypsin digestion followed by offline LC-MALDI-TOF/TOF analysis was performed, but results did not add any relevant information about the identity of the proteins. Since attempts to characterize the purified proteins were not successful, an indirect strategy was attempted. Several protein-specific antibodies were used against the 24 kDa protein. These included rabbit polyclonal antibodies to chestnut TLP (1∶30,000 dilution; 1 h; anti-TLP; kindly provided by Dr. L. Gómez, ETSIM, UPM, Madrid) or chestnut chitinases antibodies (dilution 1: 500; 1 h; anti-Chi), or with anti-complex asparagine-linked glycan serum (1∶30,000 dilution; 1 h; anti-CCD). The 24 kDa band was recognized by a pool of sera (anti-IgE) (**Figure 2B**), by anti-thaumatin-like proteins (anti-TLP) (**Figure 2C**), antibodies and by anti-carbohydrate complex determinant (anti-CCD) (**Figure 2E**). In contrast, we detected no positive recognition with other antibodies, such as antibodies produced against chestnut-chitinases (anti-Chi) (**Figure 2D**). This result suggested that a TLP could be implicated in the obeche dust sensitization, and its recognition by anti-CCD could confirm this possibility. In order to clarify this aspect, two control proteins were used: Prs a1, class I chitinase and avocado allergen; and Act d2, TLP and kiwi allergen. Both were incubated with the corresponding antibodies and with the pool of obeche sensitized sera (**Figure 2B**). The avocado-protein was not recognized by IgE, nor could we detect any positive band when the extract was incubated with anti-chitinase antibodies. However, Act d 2 was recognized by the obeche-sensitized serum pool, and a reactive band was observed when the extract was incubated with anti-TLP antibodies. Thus, the 24 kDa band may be a TLP.

### Relevance of CCD determinants in the measurement of specific IgE to obeche wood

**Figure 3:**
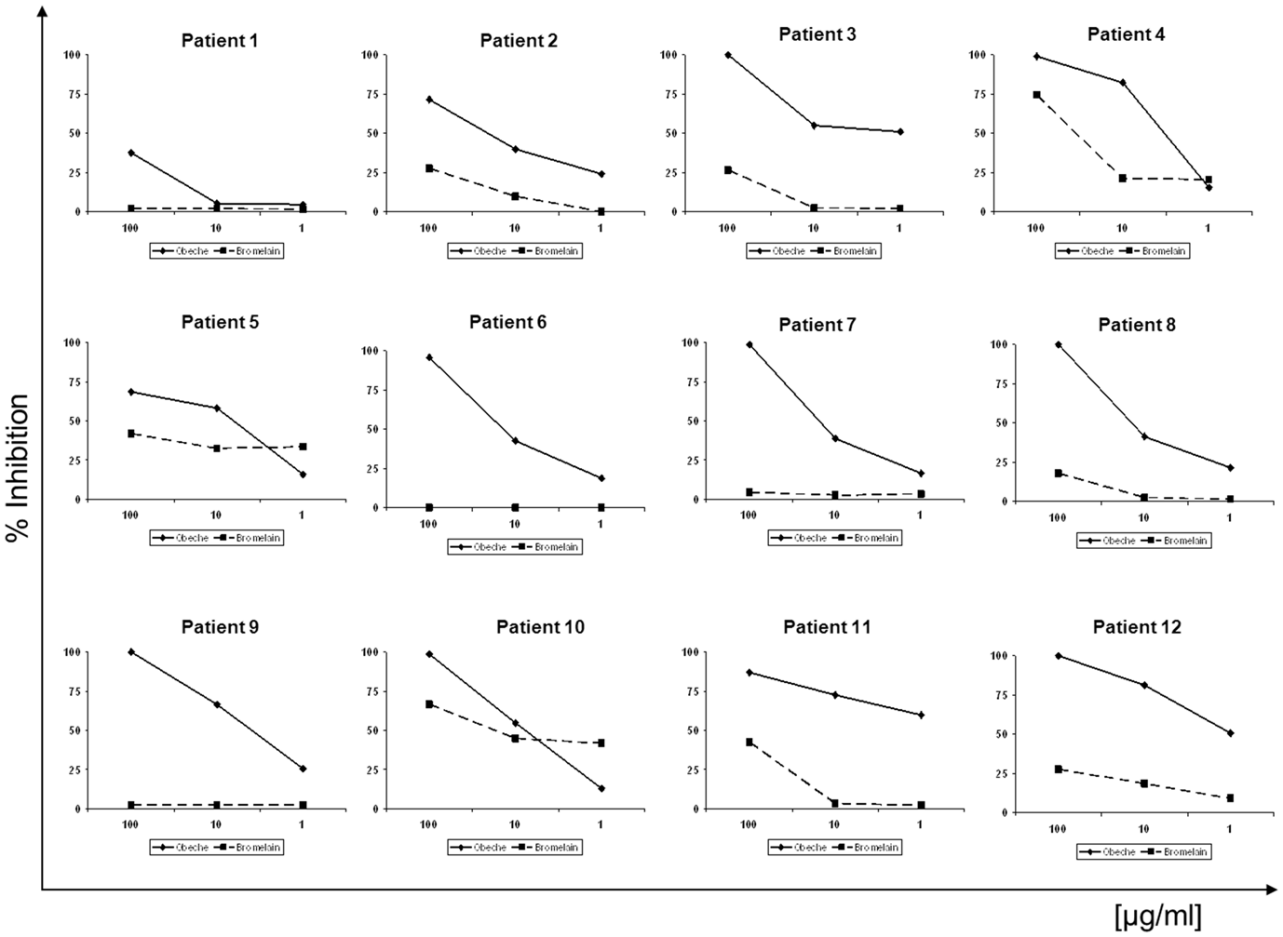
ELISA inhibition studies in ORA+ subjects. Bromelain (CCD, solid line) and obeche (dotted line) are inhibitors and obeche extract is the solid phase.

In order to determine the influence of cross-reactive carbohydrate determinants (CCDs) in the measurement of specific IgE to obeche wood in symptomatic subjects, ELISA inhibition studies were performed using obeche extract as solid phase and CCDs as inhibitors in all ORA+ patients (n = 12). Results were obtained in 11 cases, but in one case the correct interpretation of results was not possible, probably due to the low levels of IgE. A significant number of sera (9/11, 82% of cases) showed no inhibition of specific IgE binding when incubated with bromelain, but showed a specific IgE binding to obeche proteins in more than 80% of the cases. Only two sera showed significant percentage of inhibition of specific IgE binding ≥50% when incubated with bromelain (subjects #4 and 10, see **Table 2**), showing that in these two cases the positive results may be attributed to cross–reactivity with CCDs (see **Figure 3**).

**Table 2:** Results of *in vivo* and *in vitro* tests with obeche extract and purified proteins in subjects with confirmed occupational rhinitis and asthma (ORA+; R = rhinitis; R+A = rhinitis and asthma).

Subjects #	Symptoms	SPT extract	SPT 12 kDa	SPT 24 kDa	ELISA extract	ELISA 12 kDa	ELISA 24 kDa	BAT extract	BAT 12 kDa	BAT 24 kDa	+SPT grass	Food allergy history	SPT TLP-related food	SPT+ food	CCD recognition
**1**	R+A	+	−	+	+	−	+	+	+	+	+	no	−	−	n/a
**2**	R	+	nd	nd	+	+	+	nd	nd	nd	+	no	nd	nd	no
**3**	R+A	+	nd	nd	+	+	−	nd	nd	nd	+	no	nd	nd	no
**4**	R+A	+	−	−	+	−	+	+	+	+	+	no	+	Peach,chestnut,hazelnut,kiwi, melon	yes
**5**	R+A	+	−	−	+	+	+	+	+	+	−	yes	+	Peach,chestnut,apple	no
**6**	R	+	+	+	+	+	+	+	+	+	+	no	−	−	no
**7**	R	+	−	+	+	+	+	+	−	+	−	yes	+	banana,kiwi	no
**8**	R	−	−	−	+	−	+	−	−	+	+	yes	+	hazelnut	no
**9**	R	+	−	+	+	+	+	+	+	+	−	yes	−	chestnut	no
**10**	R	−	−	−	+	+	+	+	−	+	−	yes	−	−	yes
**11**	R	+	−	−	+	−	+	+	+	+	−	no	−	−	no
**12**	R	+	nd	nd	+	+	+	+	+	+	−	no	−	−	no

Results regarding food allergy assessment and CCD recognition are also included.

### Purified IgE binding proteins of obeche were recognized by exposed subjects

**Figure 4:**
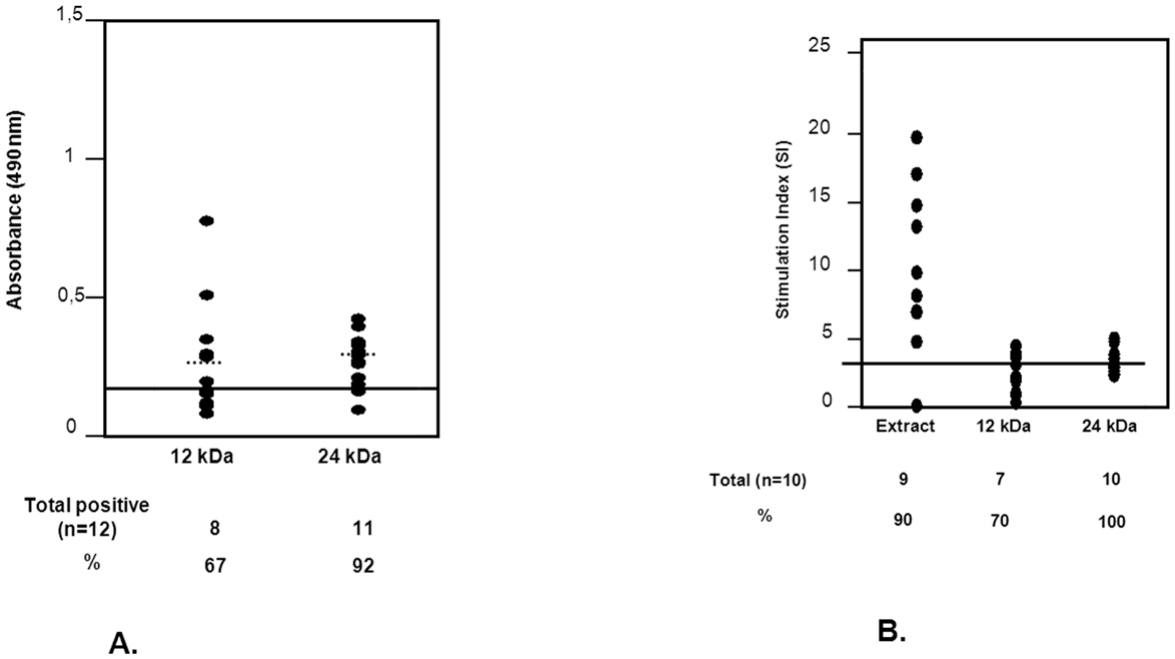
Allergenic activity of purified proteins of obeche wood dust. **4A**. IgE-binding (ELISA) to purified obeche proteins (24 and 12 kDa). Solid line shows the positivity cut-off point, dotted lines show mean OD for each group. **4B**. Basophil activation tests using the *in-house* obeche wood dust extract and purified proteins (24 and 12 kDa). The solid line shows the threshold of positivity.

Allergenic activity of purified proteins was studied by ELISA using individual sera from the 12 symptomatic subjects (**Figure 4A**). The putative TLP was recognized by 92% of the subjects, and the gamma-expansin showed a positive response in 67%. This result was confirmed when the proteins were used in BAT (**Figure 4B**) in symptomatic subjects with total extract and with purified proteins (24 and 12 kDa). A positive activation of basophils was observed in 90% of ORA+ subjects in the presence of the obeche extract. The putative TLP protein was again the most active when it was incubated with basophils from exposed subjects, being recognized by 100% of the symptomatic subjects. The 12 kDa protein showed less activation capacity (70%). The optimal concentrations were chosen based on dose-response curves and cytotoxicity studies. Results of ELISA and BAT measurements for each subject are summarized in **Table 2**. Also, results were analyzed considering whether ORA+ subjects had rhinitis or rhinitis and asthma. As shown in Table 2, subjects with rhinitis recognized more the 12 kD band by ELISA compared to subjects with asthma (75% vs.50% respectively). However, rhinitis subjects recognized less the 12 kD band by BAT than asthmatic subjects (43% vs 100%). Also, the 24 kD band was recognized similarly by both groups regarding the technique used. Therefore, there are no significant differences between both groups.

### Skin prick testing with in-house extract and purified proteins

Skin prick testing with whole obeche extract at 1 mg/ml was positive in 83% of the ORA+ subjects, with no positive responses in the non-exposed asymptomatic controls. The allergenic capacity of the obeche-purified proteins was confirmed by SPT in 9 out of 12 ORA+ workers, obtaining 44% of positive responses to the purified putative TLP protein, while 11% recognized the gamma-expansin protein. Results of SPTs for each subject are summarized in **Table 2**.

### Reactivity of SE subjects to thaumatin-containing food extracts

Since the identified 24 kDa protein was a potential TLP, as demonstrated by specific antibodies, the presence of cross-reactivity with thaumatin-containing foods and food allergy was investigated. ORA+ workers were questioned about food allergy history, and SPTs were performed with commercial banana, peach, hazelnut, chestnut, kiwi, apple and melon extracts. Almost half of ORA+ patients reported a history of food allergy (see **Table 2**). SPTs to food extracts were positive in 40% of these subjects, mostly to peach, chestnut, hazelnut and kiwi extracts.

## Discussion

Occupational asthma is the most common occupational respiratory disease in developed countries [22]. Wood dust has been identified as a risk factor for the development of asthma [23], and in some cases it is caused by IgE-mediated sensitization to protein compounds of wood dust [6]–[10], [24]–[26]. In the present study, an in-house obeche extract was produced that was recognized by SPT in 83% and by ELISA in 100% of subjects with confirmed occupational rhinitis and/or asthma, and by a small subset of asymptomatic exposed subjects (30% by ELISA).

The detailed characterization of the allergenic activity of obeche wood dust revealed two principal bands of 24 and 12 kDa, which were recognized by ELISA in 92% and 67% of the ORA+ subjects, respectively. Unfortunately, there was not a sufficient amount of protein to test the asymptomatic subjects to obtain the reactivity to these proteins in that group. As reported, bands with apparent molecular weight of 28 and 17 kDa have been identified as principal IgE-binding proteins related to obeche sensitization [6], [9], and these could correspond to our bands of 24 and 12 kDa. Surprisingly, no major IgE-binding band was observed that could be identified as Trip s1 in the present study. In addition, no recognition was shown when the in-house extract of obeche was incubated with antibodies produced against chestnut chitinase. This difference in protein recognition may be explained by several hypotheses; the first possibility is that the allergen recognition profile may differ depending on geographic characteristics, as has been described in some food allergens such as LTPs [27]. Another possible explanation is that methodological differences in dust extraction and the fact that chitinases are highly thermolabile proteins may result in the absence of 38 kDa IgE-bands in the present study and others [6], [9]–[11]. And finally, the geographical origin of the wood may influence the protein content of the extract, SDS-Page demonstrated that Trip s1 was mostly detected in wood samples coming from Ghana (*wawa*) but not in the samples coming from Cameroon (a*yous*) [28]. However, protein bands of 28 and 12 kDa were detected in dust samples from both wawa and ayous woods.

Identification of the 12 kDa protein revealed that it belonged to the gamma-expansin family [29], showing homology with tree-members of this family. Expansins refer to a family of non-enzymatic proteins found in the plant cell wall [30], and which includes small proteins grouped in two large (alpha and beta-expansins) and two small (gamma-, and epsilon- expansins) subfamilies [31]. A subset of beta-expansins has evolved a special role in grass pollen, where they are known as group 1 grass pollen allergens [21]. In contrast, the amino acid N-terminal and the trypsin-peptides obtained from the putative TLP had no correspondence with any known sequence deposited in the data bank. However, its recognition by anti-chestnut TLP antibodies suggests that it could belong to the thaumatin-like family, although more experimental analysis will be necessary to clarify its nature. Thaumatins are proteins described as plant defence proteins (PR5) against pathogen attacks and as panallergens responsible for cross-reactivity among foods and pollens [31], which may explain why some asymptomatic exposed subjects recognized the proteins mostly *in vitro* in our study [32]–[34]. For this reason, food allergy was also investigated in the ORA+ group, where 42% of the subjects reported a history of food allergy, and 40% had positive SPTs to several TLP-containing foods including peach, chestnut, hazelnut and kiwi, although the structural similarity among these TLPs and the 24 kDa protein in obeche is still unknown.

Regardless of their biochemical nature, the putative TLP and the obeche gamma-expansin were recognized by ELISA in 92% and 67% of workers with confirmed occupational rhinitis or asthma (ORA+), obtaining similar results in the BAT (100% for 24 kDa and 70% for 12 kDa protein, respectively). The results show that the protein with the highest molecular weight is the most active. However, the *in vivo* recognition was lower for both proteins, obtaining percentages of positive responses in the ORA+ group of 44% for the 24 kDa protein and only 11% for the 12 kDa protein.

The role of the cross-reactive carbohydrate determinants (CCDs) in sensitization allergy to wood proteins has been evaluated in other woods [35], showing a high rate of sensitization to CCDs in workers with *in vitro* sensitization to several woods but not among workers with a single sensitization or clinically relevant symptoms [36]. The putative thaumatin described in the present study may be glycosilated, based on the results of the incubation with anti-CCD antibodies. However, in our study, more than 80% of cases showed a specific IgE binding to obeche proteins that were not inhibited by bromelain, as demonstrated by ELISA-inhibition studies. A previous study that also described the 28 kDa as the most relevant protein in a very similar population showed that the use of a deglycosylated extract only reduced the IgE binding activity by 8.6%. Therefore, in the case of these obeche wood proteins, CCD sensitization does not seem relevant in most patients.

In conclusion, a 24 kDa putative TLP and a 12 kDa gamma-expansin were identified in obeche wood, showing a high percentage of recognition by *in vitro* studies and a modest *in vivo* recognition by subjects with confirmed rhinitis/asthma caused by obeche wood. This study will contribute to better characterize this wood, since there is a need of standardized, well-characterized extracts to correctly diagnose this disease, which is currently underestimated. Consequently, further studies are needed to establish the sensitivity and specificity parameters of these diagnostic techniques and further characterization of these and other wood allergens.
